# Preclinical evaluation of Tc-99m p5+14 peptide for SPECT detection of cardiac amyloidosis

**DOI:** 10.1371/journal.pone.0301756

**Published:** 2024-04-05

**Authors:** Stephen J. Kennel, Joseph W. Jackson, Alan Stuckey, Tina Richey, James S. Foster, Jonathan S. Wall

**Affiliations:** Department of Medicine, University of Tennessee Graduate School of Medicine, Knoxville, TN, United States of America; University of Magdeburg, GERMANY

## Abstract

**Introduction:**

Amyloid deposition is a cause of restrictive cardiomyopathy. Patients who present with cardiac disease can be evaluated for transthyretin (TTR)-associated cardiac amyloidosis using nuclear imaging with ^99m^Tc-labeled pyrophosphate (PYP); however, light chain-associated (AL) cardiac amyloid is generally not detected using this tracer. As an alternative, the amyloid-binding peptide p5+14 radiolabeled with iodine-124 has been shown to be an effective pan-amyloid radiotracer for PET/CT imaging. Here, a ^99m^Tc-labeled form of p5+14 peptide has been prepared to facilitate SPECT/CT imaging of cardiac amyloidosis.

**Method:**

A synthesis method suitable for clinical applications has been used to prepare ^99m^Tc-labeled p5+14 and tested for peptide purity, product bioactivity, radiochemical purity and stability. The product was compared with^99m^Tc-PYP for cardiac SPECT/CT imaging in a mouse model of AA amyloidosis and for reactivity with human tissue sections from AL and TTR patients.

**Results:**

The ^99m^Tc p5+14 tracer was produced with >95% yields in radiopurity and bioactivity with no purification steps required and retained over 95% peptide purity and >90% bioactivity for >3 h. In mice, the tracer detected hepatosplenic AA amyloid as well as heart deposits with uptake ~5 fold higher than ^99m^Tc-PYP. ^99m^Tc p5+14 effectively bound human amyloid deposits in the liver, kidney and both AL- and ATTR cardiac amyloid in tissue sections in which ^99m^Tc-PYP binding was not detectable.

**Conclusion:**

^99m^Tc-p5+14 was prepared in minutes in >20 mCi doses with good performance in preclinical studies making it suitable for clinical SPECT/CT imaging of cardiac amyloidosis.

## 1. Introduction

Patients seen in cardiology facilities frequently present with cardiomyopathy, and among the underlying causes of this disorder are the systemic amyloidoses [1]. The most common types of systemic amyloidosis leading to cardiac dysfunction are light chain-associated (AL) and transthyretin-associated (TTR) amyloidosis [1–6]. An amyloid-reactive peptide (p5+14), radiolabeled with iodine-124 (^124^I), has undergone clinical evaluation for the detection of multi-organ amyloid deposits using PET/CT imaging (Clinicaltrials.gov: NCT03678259) [7–10]. Data from this early phase study support use of the peptide reagent as a method for detecting amyloid in patients with AL, ATTR, and other rare forms of the disease, including identification of cardiac amyloidosis [11,12]. The ^124^I-p5+14 imaging method can only be performed in nuclear medicine facilities with PET/CT platforms which could limit the use of the radiotracer, since most cardiology facilities do not have PET/CT scanners but instead have access to SPECT or SPECT/CT imaging platforms. At present, patients suspected of having ATTR-associated cardiomyopathy are imaged with a SPECT radiotracer, ^99m^Tc-labelled pyrophosphate (PYP) [13–17], to visualize the presence of microcalcifications associated with cardiac amyloid deposits [18–20]. No consideration is given to amyloid in other organs in these studies, and this scan focuses solely on the detection of ATTR amyloid cardiomyopathy. Within the limits of the resolution of SPECT imaging these scans have been generally useful in the diagnosis of cardiac ATTR amyloidosis. However, ^99m^Tc-PYP is not an approved agent for the detection of cardiac amyloidosis. Furthermore, it fails to detect most AL cardiac amyloid [15] as does a second SPECT probe, ^123^I-labeled serum amyloid P component [21,22], which is currently used only in the UK and Netherlands.

Early diagnosis of cardiac amyloidosis can inform comprehensive and effective care of these patients [23]. A SPECT radiotracer for the sensitive and accurate detection of cardiac amyloid in patients with AL or ATTR amyloidosis that could be widely available for use in cardiology facilities would be superior to the current methods. This would provide an opportunity for cardiologists without access to PET/CT imaging capabilities to determine whether amyloidosis, of any type, was the root cause of the cardiomyopathy, and more specifically heart failure with preserved ejection fraction, thus expediting amyloid diagnosis and treatment.

A ^99m^Tc-labeled version of peptide p5+14 has been described previously and shown to detect amyloid in a murine model of systemic serum amyloid A-associated (AA) amyloidosis, secondary to chronic inflammation [24]. This product was shown to image AA amyloidosis in mice but was synthesized in small doses for animal studies. In anticipation of a clinical study with ^99m^Tc-labeled p5+14, a method was developed for synthesizing patient doses with characterization for release testing.

This work describes the preparation of a radiolabeling method for ^99m^Tc-p5+14 in 20 mCi doses which, with no purification, had >95% radiochemical yield and purity, >95% peptide purity and >90% bioactivity and could detect cardiac amyloid in a mouse model of AA amyloidosis as well as AL or ATTR amyloid deposits in human tissue sections.

## 2. Material and methods

### 2.1 Materials

All reagents were obtained in the highest grade available. NaOH and HCl were obtained from Supelco (originally from EMD Millipore Corporation (Burlington, MA)). PBS was from Intermountain Life Sciences (West Jordan, UT) and stannous chloride dihydrate was from Sigma Aldrich. Sodium monobasic phosphate was from Fischer Scientific. Peptide p5+14 (primary structure: GGGYS KAQKA QAKQA KQAQK AQKAQ AKQAK QAQKA QKAQA KQAKQ, [10] was provided by Attralus (South San Francisco, CA) and stored at -20°C as a lyophilized powder until used. The peptide was found to compromise 15% water weight and that factor was used to prepare the soluble peptide at 1.25 mg/mL in PBS, immediately prior to radiolabeling. Tc-99m sodium pertechnetate and ^99m^Tc-PYP were purchased from Cardinal Health Radiopharmacy (Knoxville, TN). All animal studies described herein were carried out in accordance with protocols approved by the University of Tennessee Institutional Animal Care and Use Committee and in accordance with the guidelines provided by the Office of Laboratory Animal Welfare (OLAW) and the Guide for the Care and Use of Laboratory Animals. The University of Tennessee Graduate School of Medicine is an Association for Assessment and Accreditation of Laboratory Animal Care International (AAALAC)-accredited institution. The use of archived human-subject–derived materials was approved by the University of Tennessee Graduate School of Medicine Institutional Review Board. Archived samples of human amyloid extract used in this study were isolated from samples obtained at autopsy from approximately 1990–2010. Samples were obtained post-mortem. Authors had access to clinical information that could identify the patients. This information, including patient name and disease type, was limited to participants engaged in this research and only provided when required.

### 2.2 Radiolabeling procedure

The peptide was labelled in alkaline solution as described by Tran et al. [25] and modified previously [24]. To a mixture containing 200 μL of 0.15 N NaOH, 800 μL of 1.25 mg/mL peptide p5+14 and 200 to 400 μL (35 mCi of ^99m^Tc04^-1^) was added 50 μL of freshly prepared 1 mg/mL of SnCl_2_·2H_2_0 in 0.01 N HCl with mixing for 2 min at room temperature. The reaction was stopped by neutralization to pH 7.4 with 65 μL of 0.5 M NaH_2_PO_4_ followed by immediate dilution to 16 mL in phosphate buffered saline. In murine experiments, 100 μg of p5+14 was radiolabeled with ^99m^Tc using the published small batch synthesis method [24].

“Kit-based” synthesis was performed by preparing lyophilized samples of the reaction components (NaOH, peptide and stannous chloride) as described for the batch synthesis except that 200 μg of stannous chloride. These were stored at -80°C under an argon atmosphere. Samples of lyophilized peptide, (with NaOH and SnCl_2_) were radiolabeled by addition of 35 mCi of ^99m^Tc04^-1^ in 1 mL of PBS and incubation for 2 min followed by dilution to a larger volume, as described above.

### 2.3 Radiotracer characterization

Radiopurity of the product, ^99m^Tc-p5+14, was tested using instant thin layer (ITLC) and reverse phase high pressure chromatographies (RP-HPLC). For ITLC, one μL of the labelled product was spotted on 8.5 x 2 cm Alumina backed SG TLC strips. The strips were developed in 50:50 methanol: 1.0 M ammonium acetate for 20 min. Strips were analyzed on a BIOSCAN ITLC reader and regions of interest values were calculated using WISCAN software. Radiolabeled p5+14 remains at the origin and free pertechnetate moves with the solvent front.

Radiopurity, peptide purity, peptide concentration and peptide identity were assessed using RP-HPLC essentially as described for ^124^I-p5+14 [24]. Samples (50 μL) were loaded on a C18 reverse phase column (Zorbax 300 SB-C18 column (USP designation L1: 300Å pore size, 45m^2^/g surface area, 2.8% carbon load; Agilent, Santa Clara, CA) and eluted with an acetonitrile, water gradient in 0.05% TFA. The peptide radioactivity eluted at 9.8 min and free pertechnetate eluted at 3.2 min. Peptide, was detected in the UV trace (Abs 215nm) at 8.6 min due to the serial positions of the UV and radioactive detectors. Peptide concentration was calculated from the absorbance profile calibrated with known peptide standards.

Bioactivity of the product was evaluated using a “pulldown” assay with synthetic AL amyloid fibrils composed of the recombinant λ6 light chain variable domain from patient WIL (rVλ6Wil fibrils) as the substrate [26]. Briefly, 25 μg of fibrils suspended in PBS containing 0.05% tween 20 (Affimetrix) were incubated with mixing for 1 h at RT with 20–40 ng of radiolabeled product. Bound and free ^99m^Tc—p5+14 were separated by centrifugation at 10,000 x g for 3 min and the % bound calculated as an index of bioactivity. Supernatants and pellets were separated after each step, and the radioactivity in each sample was measured using a Wallac 1480 gamma counter (Perkin Elmer) with a 1 min acquisition.

### 2.4 Biodistribution in an AA mouse model

Transgenic H2-L^d^-huIL-6 Balb/c mice (H2/IL-6) that constitutively express the human IL-6 transgene, were treated for induction of systemic serum amyloid protein A (AA) associated amyloidosis as previously described [7]. Briefly, 10 μg of amyloid enhancing factor (AEF), isolated by water flotation [27] from spleens of diseased mice, was injected intravenously (i.v.) in 100 μL of PBS into huIL-6 transgenic 8-wk old mice. Male and female mice were used 4–6 wk post-AEF injection and amyloid-free, strain and age-matched wild type (WT) mice served as the negative control.

For SPECT/CT imaging Tc-99m-labeled p5+14 (~200 μCi, 10 μg) or^99m^Tc—PYP (~200 μCi) was injected i.v. in 200 μL in the lateral tail vein of the mice. Animals were euthanized at 1 h or 4 h post injection by isoflurane inhalation overdose and SPECT/CT images acquired (described below) followed by necropsy. Four tissues and a blood sample were harvested from each animal, weighed in tared vials, and radioactivity quantified using a Wizard 1470 gamma spectrometer (Perkin Elmer). The % injected dose per gram (%ID/g) of tissue was calculated.

For isolated heart analyses, murine hearts were excised and perfused with a 1:1 dilution of contrast (ISOVUE 370) solution in PBS. The perfused hearts were transferred to a plastic pallet and SPECT/CT images acquired *ex vivo*. Immediately thereafter the whole organ was analyzed for residual radioactivity in the gamma spectrometer.

Image data were collected using an Inveon Trimodality SPECT/PET/CT instrument (Siemens Preclinical, Knoxville, TN). SPECT images were generated by acquiring sixty 16 second projections per revolution using 56 mm of bed travel in 1.5 revolutions and a 6-degree angle between projections. A 1.0-mm-diameter five-pinhole (Mouse Whole Body) collimator was used at 30 mm from the center of the field of view. Data were reconstructed using a Point Spread Function (PSF) model and a MAP 3D algorithm with (16 iterations and 6 subsets) with a MAP prior of 1.000 using attenuation correction and scatter correction.

CT data were acquired using an x-ray voltage biased to 80 kVp with a 500uA anode current with an aluminum filter with 0.5 mm thickness. A 200 millisecond exposure was used and 361 projections were collected covering 360 degrees of rotation on low setting with a Dalsa CT camera. The data were reconstructed using an implementation of the Feldkamp-filtered back-projection algorithm, using beam hardening correction onto a 512 x 512 x 608 matrix with an effective isotropic pixel size of 103.23 μm. The data were then down-sampled by a bin of 4. An ROI (region of interest) was drawn over the heart using IRW (Inveon Research Workplace, Siemens Molecular Imaging). The time of scan was noted for decay correction. The volume, mean, standard deviation, and max pixel values were recorded. The data were decay-corrected and cross correlated with the percent injected dose per gram from the harvested tissue samples.

### 2.5 Amyloid binding in human tissue sections

Formalin-fixed tissue sections from amyloid laden organs were deparaffinized and paraffin embedded overnight at 4°C in a solution of TBS-tween (Dako 53306) pH 7.6 with added 0.3% triton X100 (Sigma) and 3.0% BSA (Sigma). For amyloid laden heart tissues, frozen sections fixed in cold acetone were used and embedded in optimal cutting temperature compound (OCT). The sectioned tissues were rinsed in PBS/ 0.05% tween and incubated in a humidified slide box at RT with blocking solution (1 mL of stock BSA (50 mg/mL), 1 mL of stock gelatin (1%), 1.25 mL of 4 M NaCl and 6.75 mL of PBS plus 0.05% tween) for 30 min. The sections were then overlayed with ^99m^Tc-p5+14 or ^99m^Tc-PYP diluted to 60,000 cpm/10 μL in the same solution. Sections were incubated for 1 h at RT before washing for 5 min in PBS with 0.05% tween and then rinsed in water. Air dried slides were analyzed by exposure on a phosphorimager screen (multisensitive type MS) for 10 min and biodistribution of radioactivity in the tissue visualized using Cyclone phosphor imager (Perkin Elmer). The slides were then immediately dipped in NTB autoradiography emulsion (Carestream), incubated in the dark for 2 days [28] and then developed and counterstained with hematoxylin and eosin. Serial sections from the same tissue were stained with alkaline Congo red solution (0.8% w/v Congo red, 0.2% w/v KOH, 80% ethanol) for 1 h at RT followed by counterstain with Mayer’s haematoxylin for 2 min. Congo red staining of biopsied tissue samples remains the gold standard for the diagnosis of systemic amyloidosis in patients [29]. All samples were examined using a Leica DM500 light microscope fitted with cross-polarising filters (for Congo red). Digital microscopic images were acquired using a cooled CCD camera (SPOT RT-Slider; Diagnostic Instruments).

### 2.6 Pulldown experiments with human amyloid extracts

Patient tissue amyloid extracts were prepared using the water flotation method [30] and stored at room temperature as lyophilized material. In preparation for pulldown experiments, samples were weighed and suspended in PBS at 1 mg/mL, and dispersed using a polytron homogenizer, centrifuged at 13,000 x g and resuspended in PBS at the original volume. The method used for pulldown experiments was essentially as described above for WIL fibrils except that 1.0% bovine serum albumin was added to reduce background binding of ^99m^Tc-PYP.

## 3. Results

### 3.1 Preparation and characterization of ^99m^Tc-p5+14

**Fig 1:**
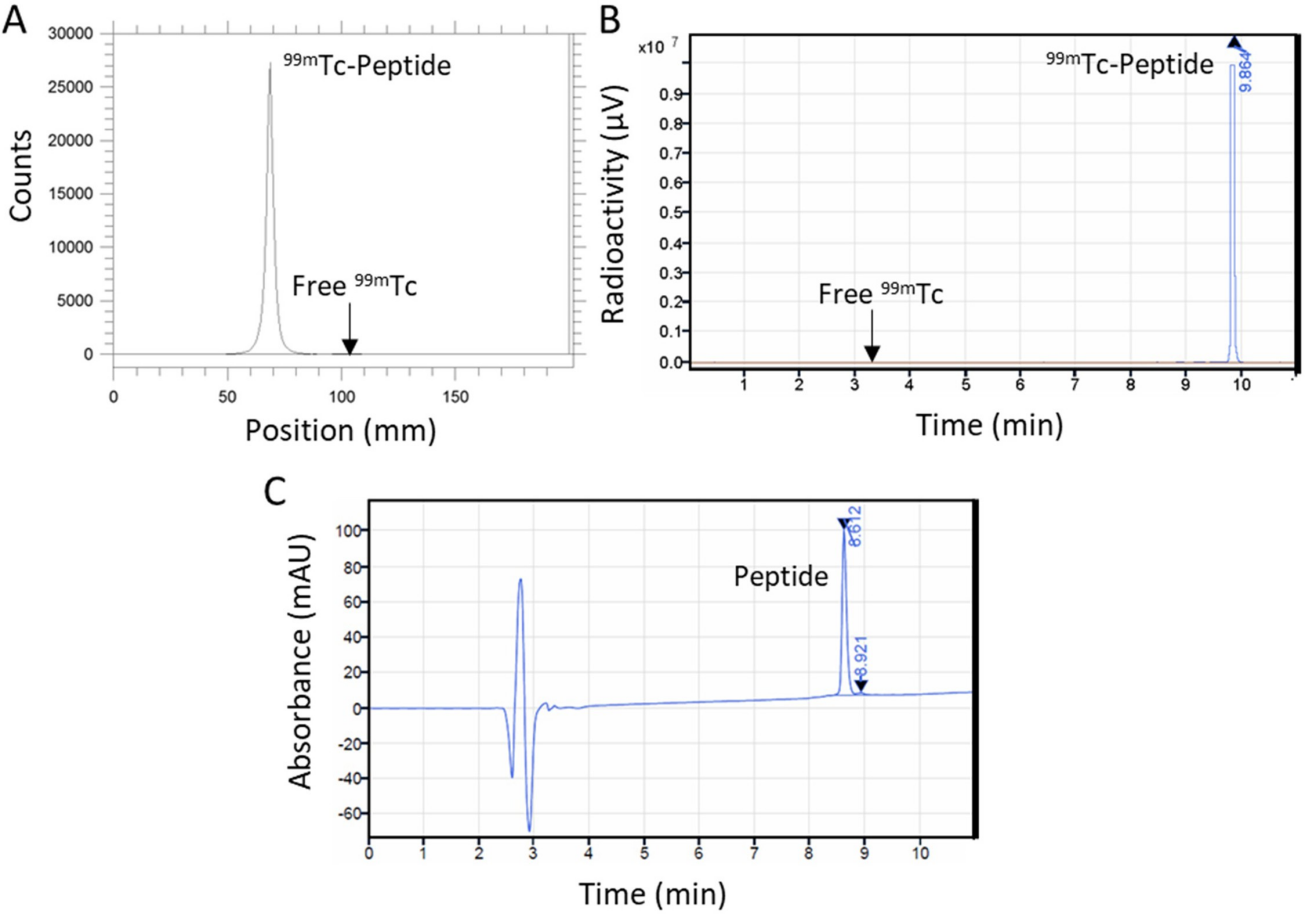
Chromatographic characterization of ^99m^Tc p5+14. A 35 mCi reaction was performed and the product evaluated for radiopurity by ITLC (A), RP-HPLC (B). The peptide purity and identity were assessed by RP-HPLC from the same run using the A215 nm detector (C).

Five independent batches of ^99m^Tc-p5+14 were synthesized. In each one, the reaction product immediately following synthesis (time = 0 h) was found to have a radiochemical yield and radiochemical purity of >95% (**Fig 1A and 1C)** and bioactivity of >90% (**Table 1**). Peptide purity and identity, established using RP-HPLC, were >95% (**Table 1 and Fig 1B**). The major peptide impurity eluted as a small shoulder on the trailing side of the main peptide peak at 8.921 min (**Fig 1C**). Samples were tested after storage at RT for 3 h retained a radiochemical yield and purity of >95%. However, some loss of ^99m^TcO_4_^-1^ from the peptide was observed at 6 h post synthesis (**Table 1**).

**Table 1:** Radiopurity, peptide purity and bioactivity of ^99m^Tc-p5+14.

Time post-synthesis (h)	Radiopurity—ITLC (%)	Radiopurity–HPLC (%)	Peptide purity (AUC)	Bioactivity (bound %)
**0**	99.0±0.3	>99.0	97.8±0.8	94.0±0.2
**3**	98.1±0.4	>99.0	97.5±1.6	92.9±0.1
**6**	85.4±1.7	63.4±1.1	91.3±5.3	72.2±0.2

± indicates standard deviation of 5 separate preparations.

### 3.2 Biodistribution in AA and WT mice

**Fig 2:**
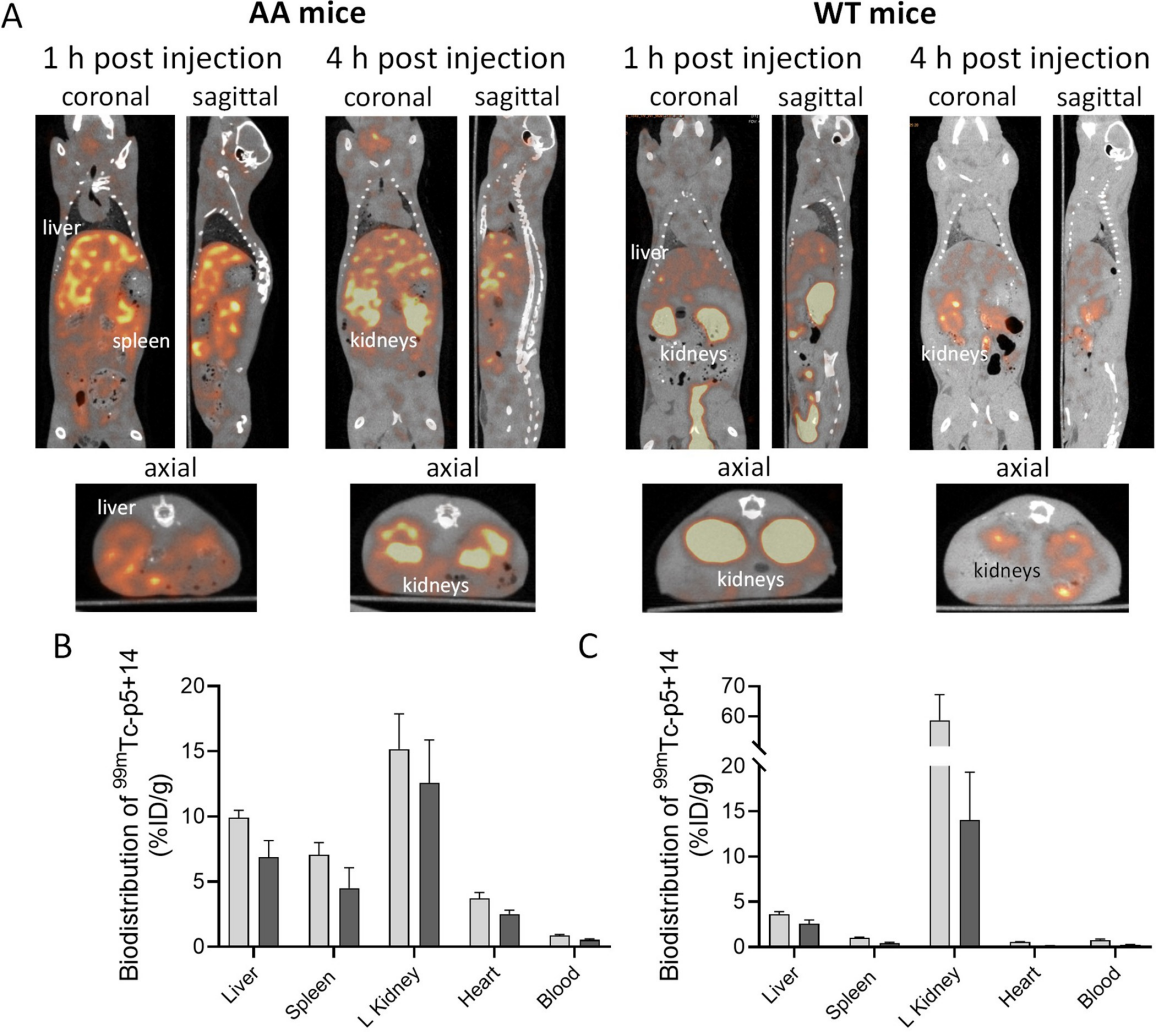
Biodistribution of ^99m^Tc-p5+14 in AA and WT mice. The biodistribution of ^99m^Tc-p5+14 (~100 μCi, ~20 μg) administered intravenously in mice with systemic AA amyloidosis or WT controls (n = 3) was assessed by small animal SPECT/CT imaging (A) at 1 or 4 h post injection. Uptake of ^99m^Tc-p5+14 in the heart, liver, spleen, kidney, and blood was quantified in mice with AA amyloidosis (B) and wild type controls (C) at 1 (light bars) and 4 hours (dark bars) after imaging and expressed as percent injected dose per gram of tissue (%ID/g).

The ^99m^Tc-p5+14 was rapidly taken up in liver and spleen of mice with severe systemic AA amyloidosis, the organs with the highest amyloid burden, as evidenced in SPECT/CT images (**Fig 2A**). Retention of the hepatosplenic uptake persisted for at least 4 h pi (Fig 2). Radioactivity in the kidney, through which the peptide is cleared, was visible over the 1–4 h pi (kidneys are not visible in the 1 h AA mouse SPECT/CT coronal slice, **Fig 2**, which focuses on the liver and spleen). In WT mice the organ with greatest activity in the SPECT/CT images, at both times pi, is the kidney, the organ of clearance. Diffuse blood pool radioactivity was seen in the liver of WT at 1 h pi but, in contrast to mice with AA amyloidosis, no significant radiotracer retention was observed in the liver or spleen at 4 h pi.

Quantitative analysis of the ^99m^Tc-p5+14 biodistribution (**Fig 2B and 2C**) indicated <3% ID/g of the radiotracer in the liver and spleen of WT mice while radioactivity in the kidney at 1 h pi was 60% ID/g, which decreased at 4 h pi to ~15% ID/g. In contrast, radiotracer was evident in the liver, spleen, and heart of AA mice at levels roughly 3 times those in WT mice at either time point. Conversely, evident radioactivity in the kidneys of AA mice did not exceed 15% ID/g (Fig 2B).

**Fig 3:**
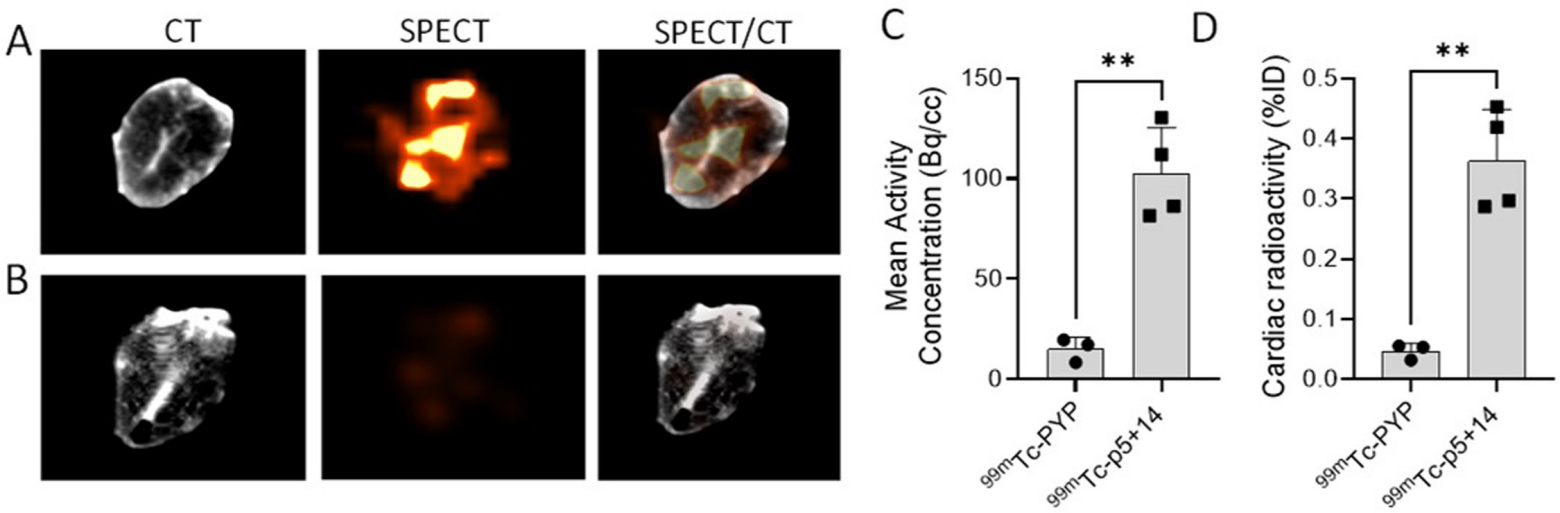
Detection of ^99m^Tc-p5+14 and ^99m^Tc-PYP in the heart of mice with AA amyloidosis. Transgenic mice (n = 3–4) with AA amyloidosis, at 6 wk post-AEF, were administered ~200 μCi of either ^99m^Tc p5+14 (A) or ^99m^Tc-PYP (B) and the hearts were excised 1 hour thereafter, perfused with CT contrast agent diluted 1:1 (v/v) in PBS, and imaged by SPECT/CT. There was significantly greater uptake of ^99m^Tc-p5+14 in the heart (*p*<0.01) assessed by manual image analysis (C) and gamma counting (D).

Cardiac uptake of the radiotracers was examined by SPECT/CT imaging of the heart excised from mice euthanized at 1 h pi **(Fig 3)**. Regions of interest were manually drawn around the whole heart in each scan and the radioactivity per unit volume (Bq/mL) measured. The mean activity concentration for ^99m^Tc-PyP and ^99m^Tc-p5+14 in the heart (**Fig 3B**) was shown to be 15.1 Bq/cc ± 4.5 (n = 3) and 102 Bq/cc ±18.6 (n = 4), respectively (**p = 0.0014). The mean retention of ^99m^Tc-PYP and ^99m^Tc-p5+14 in the heart quantified using a gamma counter (**Fig 3C**) was 0.047%ID/g ± 0.01 and 0.36%ID/g ± 0.07, respectively (**p = 0.0015).

### 3.3 Binding of ^99m^Tc-p5+14 to human AL and ATTR amyloid

**Fig 4:**
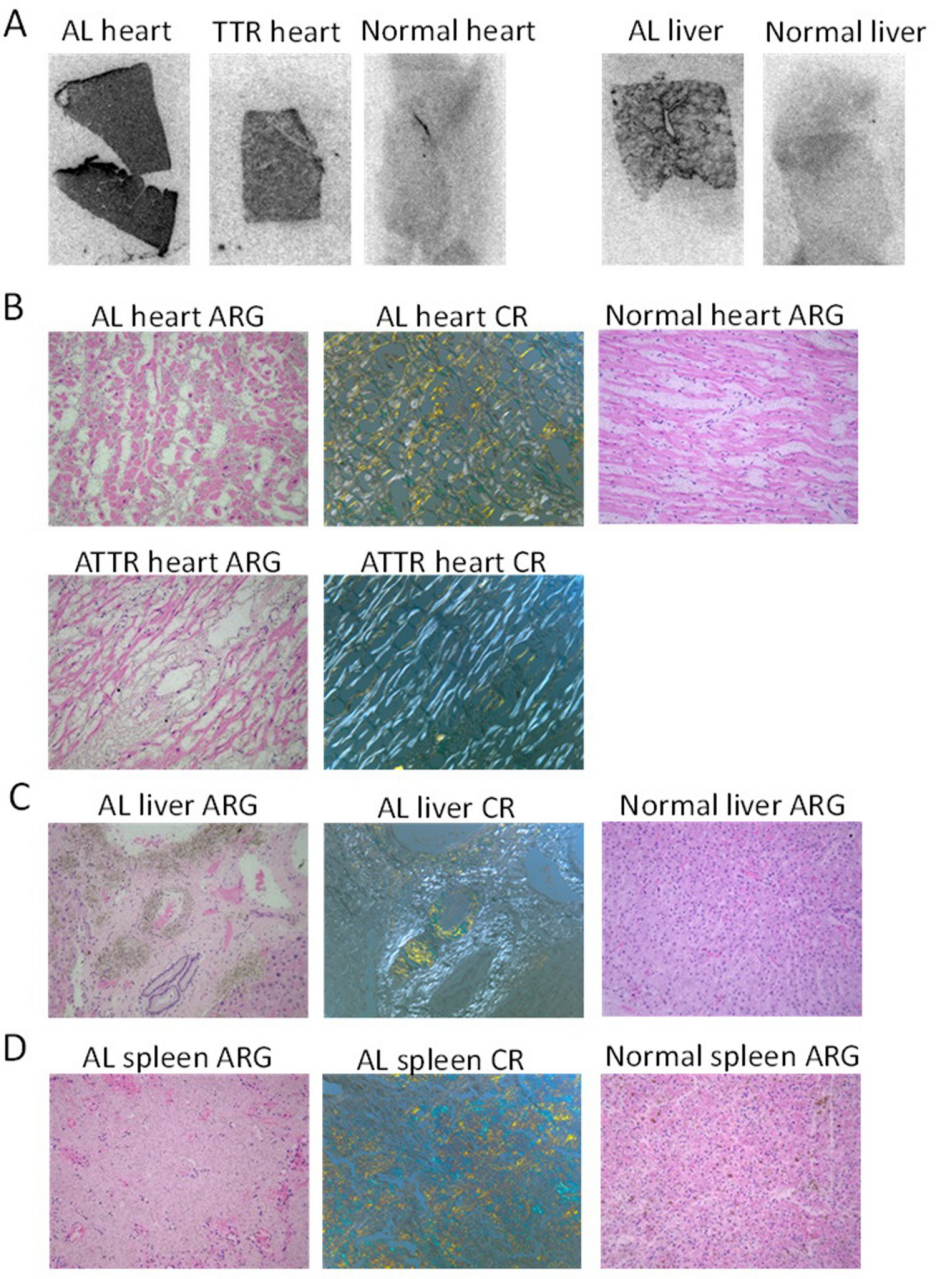
Binding of ^99m^Tc-p5+14 to human AL and ATTR amyloid in formalin fixed tissue sections. Formalin-fixed human amyloid-laden tissue sections were overlayed with ^99m^Tc p5+14 (~60 nCi, ~ 7 ng in 10 μL), incubated and washed as described in methods. (A) Slides were analyzed by phosphorimaging for 10 min before processing for autoradiography for 2 days (B–D). Binding of the radiolabeled peptide to amyloid was evidenced by the presence of black silver grains in the phosphor images and autoradiographs (ARG). The presence of higher density grains correlated with amyloid deposits seen as green gold birefringent material in Congo red (CR)-stained consecutive tissue sections. No binding of the peptide was seen in sections of normal heart, liver, or spleen tissue. Images were acquired at 20x magnification.

To demonstrate that the ^99m^Tc-p5+14 probe could bind to human amyloid, sections of formalin-fixed tissues containing AL and ATTR amyloid deposits were overlayed with a solution of the radiotracer. Phosphorimaging of the tissue samples demonstrated significantly more binding of ^99m^Tc-p5+14 in amyloid-laden organs as compared to normal tissues (**Fig 4A**). To visualize the radiotracer distribution at higher resolution microautoradiography was performed. The distribution of radioactivity on the tissues corresponded to that of amyloid deposits as detected by Congo red staining of consecutive tissue sections (**Figs 4B–4D and S1**).

**Fig 5:**
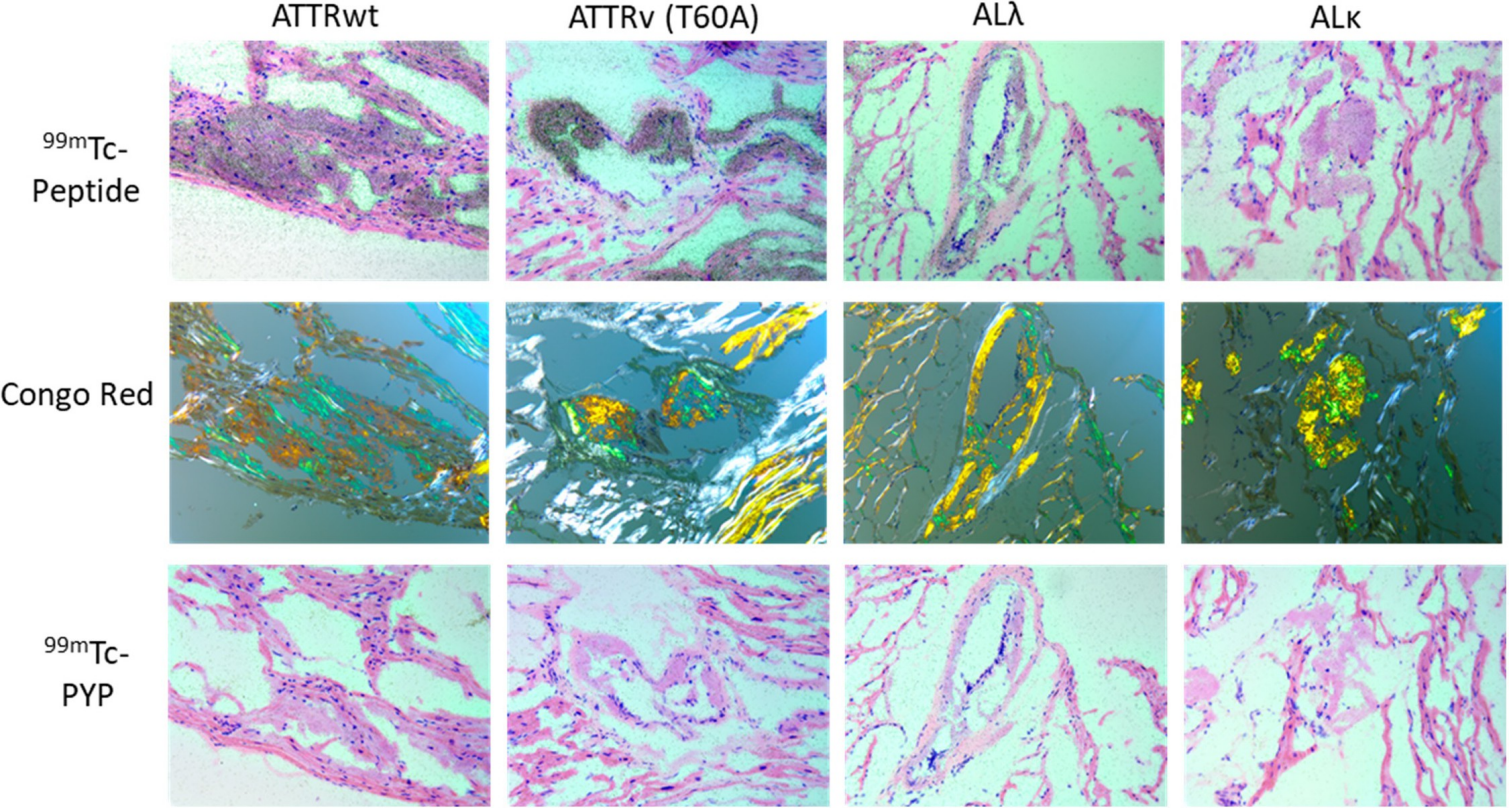
Binding of ^99m^Tc-p5+14 to human AL and ATTR cardiac amyloid in fresh tissue sections. Fresh frozen, acetone-fixed human AL or ATTR amyloid-laden tissue sections were overlayed with ^99m^Tc p5+14 (upper panels) or ^99m^Tc-PYP (bottom panels) incubated, washed, and processed for autoradiographic imaging for 2 days. Binding of the radiolabeled peptide to amyloid was evidenced by the presence of black silver grains in the autoradiographs (top and bottom panels). The presence of higher density grains correlated with amyloid deposits seen as green gold birefringent material in Congo red-stained consecutive tissue sections (middle panels). Images were acquired using a 20x objective magnification.

Fresh frozen sections of amyloid-laden heart tissue from five patients with ATTR or AL amyloidosis were similarly evaluated by microautoradiography using either ^99m^Tc-PYP or ^99m^Tc-p5+14. Uptake of ^99m^Tc-p5+14 in Congo red-positive amyloid deposits was readily demonstrable (**Fig 5, top panels**) while no specific uptake of ^99m^Tc-PYP was seen using this technique in either the AL or ATTR amyloid containing samples (**Fig 5, bottom panels**).

To further compare the amyloid reactivity of ^99m^Tc-PYP and ^99m^Tc-p5+14 patient extracts of AL, ATTRv, or ATTRwt amyloid were used as substrates in pulldown experiments (**Table 2**). Values ranging from 49% to 93% were obtained for the binding of ^99m^Tc-p5+14 with ATTRv, ATTRwt and AL amyloid extracts, whereas no specific binding of ^99m^Tc-PYP (<4%) was observed. The specificity of ^99m^Tc-p5+14 binding was further demonstrated by incubation with murine splenic AA amyloid extract in the presence of 20 μg non-radiolabeled peptide where 68% peptide binding on the AA extract was reduced to 4% in the presence of the excess unlabeled competitor peptide.

**Table 2:** Binding of ^99m^Tc-p5+14 and ^99m^Tc-PYP to patient amyloid extracts.

Amyloid type	Tissue	^99m^Tc-p5+14 bound (%)	^99m^Tc-PYP bound (%)
**ALκ**	Liver	93.3±0.1	1.5±0.1
**ALλ**	Spleen	88.1±6.2	1.3±0.5
**ATTRv (T60A)**	Heart	83.4±1.6	1.3±0.0
**ATTRv (T60A)**	Heart	49.8±4.6	1.6±0.3
**ATTRv**	Heart	79.0±0.2	1.0±0.2
**ATTRwt**	Heart	85.9±0.1	2.7±0.0
**ATTRwt**	Heart	89.4±1.7	6.3±1.3
**rVλ6WIL fibrils**	Synthetic	91.1±0.2	1.5±0.3
**Murine AA extract**	Spleen	67.8±1.1	nd*

* not detected.

To determine if this method of ^99m^Tc p5+14 synthesis was amenable to kit production, samples containing NaOH, peptide and stannous chloride were lyophilized and stored at -80°C after purging with argon gas. Samples of lyophilized peptide p5+14/NaOH/SnCl_2_ were radiolabeled by addition of 35 mCi of ^99m^TcO_4_^-1^ in 1 mL of PBS and incubation for 2 min followed by dilution as described for the standard method. These lyophilized “kit” preparations used at day 7, 98 and 128 days post preparation gave radiochemical yield and radiopurity of 96.9%, 95.8%, and 96.8% purity, respectively; however, the bioactivity of these samples was decreased with time as compared to non-kit synthesis methods (Day 7 = 76.8%; Day 98 = 75.1%; and Day 128 = 75.2% bound).

## 4. Discussion

Accurate and rapid diagnosis of systemic amyloidosis remains challenging due to the diverse symptomology, limitations in the accuracy of current diagnostic tests, as well as a lack of disease awareness among physicians [23]. We have developed poly cationic peptides that bind specifically to many if not all types of amyloid. One of these (p5+14) radiolabeled with the positron emitter iodine-124 has been evaluated in a phase 1/2 clinical trial for amyloid imaging by PET/CT imaging (Clinicaltrials.gov: NCT03678259). This radiotracer can effectively detect amyloid deposits, notably with high sensitivity for cardiac amyloid, in patients with diverse types of amyloidosis as well as well as extracardiac amyloid throughout the body [9,31].

Cardiac amyloidosis, in particular, results in significant mortality and morbidity with time to death post-diagnosis ranging from 9 months (AL) to 3 years (ATTR) [32]. Several underlying pathologies can lead to cardiac hypertrophy with preserved ejection fraction, characteristic of amyloidosis, thus a facile method to test for amyloid as the etiology could improve early and accurate diagnosis of amyloidosis and initiation of appropriate treatment. The need and benefits of early diagnosis of amyloidosis are well documented [23,33]. Currently, the most common method for detecting cardiac amyloid is SPECT/CT or planar imaging with the Tc-99m labeled bone seeking agents (*e*.*g*., PYP and 3,3-diphosphono-1,2-propanodicarboxylic acid [DPD]). ^99m^Tc-PYP detects calcium deposits associated with ATTR-associated cardiac amyloid, and is not effective for detecting AL deposits [34]. The utility of the ^99m^Tc-PYP scan method has been recently reviewed [17]. The early phase PET/CT imaging clinical evaluation of ^124^I p5+14 has demonstrated the potential to detect both AL and ATTR cardiac amyloid, as well as other types (e.g. in patients with AApoA1 and ALys) [9,31]. The Aβ amyloid binding PET probe [^18^F]-florbetapir is also effective at detecting cardiac amyloid but is not bound stably by cardiac ATTR deposits and cannot reliably image hepatic or renal AL amyloid [35].

Although PET/CT of amyloid load is valuable, due to its ability to provide high-resolution, quantitative whole-body images, it can be relatively expensive and requires access to a PET/CT or PET/MR imaging system. Many cardiology clinics have access to dedicated SPECT or SPECT/CT platforms, which are routinely used to evaluate myocardial perfusion and which can also be used to assess amyloid-associated cardiomyopathy in patients. As an alternative to the radioiodinated form, ^99m^Tc-p5+14 could be effectively used to detect all forms of cardiac amyloid in the community cardiology setting.

We have previously radiolabeled peptide p5+14 with Tc-99m at small scale using a “wet chemistry” method [24] and the current study expands on these observations. The optimized synthesis method described herein generates radiolabeled product of high purity and bioactivity that detects AA amyloidosis in the major organs (other than kidney) in mice (**Figs 2 and 3**). These data are comparable to those previously published for ^125^I p5+14 [10] as well as ^99m^Tc-p5+14 produced by wet chemistry and purified by gel filtration [24].

Importantly, this probe does not bind significantly to normal tissue and is detected in highest concentrations in the kidneys of healthy mice due to clearance of the unbound peptide *via* renal excretion. This suggests that detecting renal amyloidosis using the ^99m^Tc variant of this peptide may be challenging, but, notably, can be achieved using the radioiodinated variant [10]. The ^99m^Tc p5+14 radiotracer could be an important tool for the detection of cardiac amyloidosis of any type in the community cardiology setting or wherever ready access to a PET/CT platform is problematic.

A particular focus of this work is the dedicated use of this radiotracer for the detection of cardiac amyloid. Our data indicate that ^99m^Tc-p5+14 can detect cardiac amyloid when injected intravenously into AA mice. *Ex vivo* imaging was used to detect heart uptake due to the large accumulation of probe in hepatic amyloid and the proximity of this organ to the heart, making the detection of scant cardiac AA amyloid deposits seen in this mouse model difficult to appreciate in whole body images. We anticipate that the significant amyloid deposits in the hearts of patients with AL and ATTR amyloidosis and the improving resolution of commercial SPECT/CT instruments will readily permit detection of cardiac amyloid in patients even in the presence of significant amyloid load in other abdominothoracic organs. Comparing cardiac uptake of ^99m^Tc-p5+14 with ^99m^Tc-PYP, in excised hearts of mice with AA amyloid, showed that ^99m^Tc-p5+14 accumulated approximately 8-fold higher than ^99m^Tc-PYP in the organs (**Fig 3)**. It is possible that calcifications in murine cardiac AA amyloid are insufficient for detection by PYP; although human cardiac AA does image with bone-seeking agents, albeit infrequently [36].

Small scale preparation of ^99m^Tc-p5+14 batches for murine experiments is not appropriate for patient dosages and requires post-labelling purification. After preliminary testing of reagent concentrations and reaction times, an optimal method for preparing larger batches suitable for human imaging was developed. These studies were performed with reagents and quality assurance methods suitable for use in early phase clinical studies. We have prepared ^99m^Tc-p5+14 at specific activities of ~35 mCi/ mg peptide which would be sufficient for a patient SPECT/CT dose. The product was prepared in high radiochemical yield, radiochemical and peptide purity, and bioactivity directly from the reaction mixture without purification (**Fig 1**) and showed excellent stability characteristics when stored at RT in an administration syringe, for at least 3 h post-synthesis.

We demonstrated that the ^99m^Tc-p5+14 reagent generated using this method bound human amyloid deposits of diverse type in liver, spleen, and heart tissues. Specifically, phosphorimaging and autoradiography studies (**Fig 5**) of both AL and ATTR amyloid-containing tissues bound the radiotracer, whereas normal tissue does not. Microautoradiographic evaluation of formalin-fixed or frozen tissue sections (**Figs 4 and 5**) demonstrated specific localization of the radioactivity at sites in the tissues consistent with the presence of amyloid deposits as demonstrable by Congo red staining. Frozen heart tissue sections were used to compare ^99m^Tc-PYP and ^99m^Tc-p5+14 using an overlay method. No specific binding of ^99m^Tc-PYP to these samples was detected by microautoradiography (**Fig 5**). It is possible that the ATTR amyloid tissue sections used in this study did not contain sufficient calcium deposits for detection by ^99m^Tc-PYP. Similarly, pulldown experiments using human amyloid extracts demonstrated that ^99m^Tc-p5+14 bound with high efficiency and specificity with structurally complex amyloid extracts and synthetic fibrils (**Table 2**), whereas ^99m^Tc-PYP did not. These data parallel and extend preclinical study results of the Tc-99m- and I-124-labeled p5+14 probes and indicate that ^99m^Tc-p5+14 will be an effective radiotracer for detecting cardiac amyloid by SPECT/CT or similar gamma imaging modality.

To optimize the ^99m^Tc labelling procedure for compatibility with commercial applications, a dried, radiolabeling “kit” was generated and evaluated. Many such kits are available for other ^99m^Tc-labeled radiotracers [37]. However, they all utilize neutral pH labelling conditions for coupling ^99m^Tc to established chelators such as 6-hydrazinonicotinic acid (HYNIC) with or without triglycine. Labelling of p5+14 was conducted at alkaline pH>11 using a modified method of Tran *et al*. (25). Excellent labelling was achieved with the high pH formulated kits even after storage for 3 months. Furthermore, the reaction conditions yielded product that required no purification, although bioactivity was decreased in these products from 93% to ~80%. Further refinement of the kit components including stabilization of the stannous chloride will be necessary to optimize this labeling methodology.

The structure of the ^99m^Tc-p5+14 product is not known. We hypothesize that the chelator-free incorporation of ^99m^Tc into p5+14 results from a conformational arrangement at the N terminus of the peptide involving coordination of the three N terminal glycine residues and possibly one (or more) of the N terminal lysine side chains. Some support for this speculation is that triglycine moieties are known to be active ^99m^Tc chelators (37) and that labeling of ^99m^Tc p5+14 does not take place at neutral pH where secondary structure remains unperturbed. Effective incorporation of Tc-99m into p5+14 using our method occurs at a pH above the pK of the α-amino group (10.5) and pI (9.74) of lysine. Moreover, conditions such as boiling in SDS-PAGE loading buffer that denature the ^99m^Tc-p5+14 structure result in ^99m^Tc release. As with most Tc-99m probes, strong reducing agents like mercaptoethanol or dithiothreitol cause release of the ^99m^Tc from the peptide likely in a form of ^99m^Tc oxides. Nonetheless, the data show that the labelled product retains stability for at least 3 h when adjusted to neutral pH and diluted in PBS.

## 5. Conclusion

This paper describes a facile method for radiolabeling the pan-amyloid reactive peptide p5+14 with ^99m^Tc0_4_^-1^ to yield a product suitable for the detection of amyloid by SPECT imaging. This radiotracer could be readily used in the community cardiology setting as well as Nuclear Medicine Depts. for the detection and diagnosis of cardiac amyloidosis using SPECT or SPECT/CT imaging.

## Supporting information

S1 Fig Formalin-fixed AL amyloid-laden kidney tissue sections were overlayed with ^99m^Tc p5+14 (~60 nCi, ~ 7 ng in 10 μL), incubated and washed as described in methods. Slides were processed for autoradiography for 2 days. Binding of the radiolabeled peptide to amyloid was evidenced by the presence of black silver grains in the autoradiographs (ARG). The presence of higher density grains correlated with amyloid deposits seen as red fluorescence in the Congo red (CR)-stained consecutive tissue sections.
(DOCX)

S1 Data (XLSX)

## References

[1] Muchtar E, Dispenzieri A, Magen H, Grogan M, Mauermann M, McPhail ED, et al. Systemic amyloidosis from A (AA) to T (ATTR): a review. J Intern Med. 2020. 10.1111/joim.13169 32929754

[2] Bart NK, Thomas L, Korczyk D, Atherton JJ, Stewart GJ, Fatkin D. Amyloid Cardiomyopathy. Heart Lung Circ. 2020;29(4):575–83. doi: 10.1016/j.hlc.2019.11.019 32001152

[3] Falk RH, Alexander KM, Liao R, Dorbala S. AL (Light-Chain) Cardiac Amyloidosis: A Review of Diagnosis and Therapy. J Am Coll Cardiol. 2016;68(12):1323–41. doi: 10.1016/j.jacc.2016.06.053 27634125

[4] Kristen AV. Amyloid cardiomyopathy. Herz. 2020;45(3):267–71. 32107564 10.1007/s00059-020-04904-4

[5] Ruberg FL, Grogan M, Hanna M, Kelly JW, Maurer MS. Transthyretin Amyloid Cardiomyopathy: JACC State-of-the-Art Review. J Am Coll Cardiol. 2019;73(22):2872–91. doi: 10.1016/j.jacc.2019.04.003 31171094 PMC6724183

[6] Wolfson AM, Shah KS, Patel JK. Amyloid and the Heart. Curr Cardiol Rep. 2019;21(12):164. doi: 10.1007/s11886-019-1230-9 31792619

[7] Martin EB, Williams A, Richey T, Stuckey A, Heidel RE, Kennel SJ, et al. Comparative evaluation of p5+14 with SAP and peptide p5 by dual-energy SPECT imaging of mice with AA amyloidosis. Sci Rep. 2016;6:22695. doi: 10.1038/srep22695 26936002 PMC4776142

[8] Wall JS, Kennel SJ, Martin EB. Dual-Energy SPECT and the Development of Peptide p5+14 for Imaging Amyloidosis. Mol Imaging. 2017;16:1536012117708705. doi: 10.1177/1536012117708705 28654386 PMC5469514

[9] Wall JS, Martin EB, Endsley A, Stuckey AC, Williams AD, Powell D, et al. First in Human Evaluation and Dosimetry Calculations for Peptide (124)I-p5+14-a Novel Radiotracer for the Detection of Systemic Amyloidosis Using PET/CT Imaging. Mol Imaging Biol. 2022;24(3):479–88. doi: 10.1007/s11307-021-01681-2 34786667

[10] Wall JS, Martin EB, Richey T, Stuckey AC, Macy S, Wooliver C, et al. Preclinical Validation of the Heparin-Reactive Peptide p5+14 as a Molecular Imaging Agent for Visceral Amyloidosis. Molecules. 2015;20(5):7657–82. doi: 10.3390/molecules20057657 25923515 PMC4442108

[11] Stuckey A, Martin EB, Powell D, Fu Y, Besozzi M, Hall S, et al. Time resolved biodistribution of peptide 124I-p5+14 in patients with systemic AL amyloidosis. Journal of Nuclear Medicine. 2020;61(Supp 1):3127.

[12] Wall JS, Stuckey A, Martin EB, Richey T, Williams A, Wooliver C, et al. Preliminary Phase 1 Data on the Safety and Efficacy of a Novel PET Radiotracer, 124I-p5+14, for Imaging Systemic Amyloidosis. Blood. 2019;134(Supp 1):3034.

[13] Bokhari S, Castaño A, Pozniakoff T, Deslisle S, Latif F, Maurer MS. (99m)Tc-pyrophosphate scintigraphy for differentiating light-chain cardiac amyloidosis from the transthyretin-related familial and senile cardiac amyloidoses. Circ Cardiovasc Imaging. 2013;6(2):195–201. doi: 10.1161/CIRCIMAGING.112.000132 23400849 PMC3727049

[14] Bokhari S, Morgenstern R, Weinberg R, Kinkhabwala M, Panagiotou D, Castano A, et al. Standardization of (99m)Technetium pyrophosphate imaging methodology to diagnose TTR cardiac amyloidosis. J Nucl Cardiol. 2018;25(1):181–90. doi: 10.1007/s12350-016-0610-4 27580616

[15] Castano A, Haq M, Narotsky DL, Goldsmith J, Weinberg RL, Morgenstern R, et al. Multicenter Study of Planar Technetium 99m Pyrophosphate Cardiac Imaging: Predicting Survival for Patients With ATTR Cardiac Amyloidosis. JAMA Cardiol. 2016;1(8):880–9. doi: 10.1001/jamacardio.2016.2839 27557400

[16] Hanna M, Ruberg FL, Maurer MS, Dispenzieri A, Dorbala S, Falk RH, et al. Cardiac Scintigraphy With Technetium-99m-Labeled Bone-Seeking Tracers for Suspected Amyloidosis: JACC Review Topic of the Week. J Am Coll Cardiol. 2020;75(22):2851–62. doi: 10.1016/j.jacc.2020.04.022 32498813

[17] Nichols KJ, Yoon SY, Van Tosh A, Palestro CJ. (99m)Tc-PYP SPECT and SPECT/CT quantitation for diagnosing cardiac transthyretin amyloidosis. J Nucl Cardiol. 2022. doi: 10.1007/s12350-022-03133-y 36352087

[18] Khor YM, Cuddy SAM, Singh V, Falk RH, Di Carli MF, Dorbala S. (99m)Tc Bone-Avid Tracer Cardiac Scintigraphy: Role in Noninvasive Diagnosis of Transthyretin Cardiac Amyloidosis. Radiology. 2023;306(2):e221082. doi: 10.1148/radiol.221082 36537896 PMC9885342

[19] Stats MA, Stone JR. Varying levels of small microcalcifications and macrophages in ATTR and AL cardiac amyloidosis: implications for utilizing nuclear medicine studies to subtype amyloidosis. Cardiovasc Pathol. 2016;25(5):413–7. doi: 10.1016/j.carpath.2016.07.001 27469499

[20] Yamagata N, Fujio J, Hirai R, Matsumaru M, Tanimura S, Inokuchi C, et al. Marked hepatomegaly due to AA type amyloidosis in a case with Castleman’s disease. Int J Hematol. 2006;84(1):70–3. doi: 10.1532/IJH97.05126 16867906

[21] Hachulla E, Maulin L, Deveaux M, Facon T, Bletry O, Vanhille P, et al. Prospective and serial study of primary amyloidosis with serum amyloid P component scintigraphy: from diagnosis to prognosis. Am J Med. 1996;101(1):77–87. doi: 10.1016/s0002-9343(94)00054-9 8686719

[22] Hazenberg BP, van Rijswijk MH, Piers DA, Lub-de Hooge MN, Vellenga E, Haagsma EB, et al. Diagnostic performance of 123I-labeled serum amyloid P component scintigraphy in patients with amyloidosis. Am J Med. 2006;119(4):355 e15-24. doi: 10.1016/j.amjmed.2005.08.043 16564782

[23] Kittleson MM, Ruberg FL, Ambardekar AV, Brannagan TH, Cheng RK, Clarke JO, et al. 2023 ACC Expert Consensus Decision Pathway on Comprehensive Multidisciplinary Care for the Patient With Cardiac Amyloidosis: A Report of the American College of Cardiology Solution Set Oversight Committee. J Am Coll Cardiol. 2023;81(11):1076–126. 36697326 10.1016/j.jacc.2022.11.022

[24] Kennel SJ, Stuckey A, McWilliams-Koeppen HP, Richey T, Wall JS. Tc-99m Radiolabeled Peptide p5 + 4 is an Effective Probe for SPECT Imaging of Systemic Amyloidosis. Mol Imaging Biol. 2016;18(4):483–9. 26573301 10.1007/s11307-015-0914-9 PMC4874933

[25] Tran T, Engfeldt T, Orlova A, Widström C, Bruskin A, Tolmachev V, et al. In vivo evaluation of cysteine-based chelators for attachment of 99mTc to tumor-targeting Affibody molecules. Bioconjug Chem. 2007;18(2):549–58. doi: 10.1021/bc060291m 17330952

[26] Wall J, Schell M, Murphy C, Hrncic R, Stevens FJ, Solomon A. Thermodynamic instability of human lambda 6 light chains: correlation with fibrillogenicity. Biochemistry. 1999;38(42):14101–8. doi: 10.1021/bi991131j 10529258

[27] Kaplan B, Murphy CL, Ratner V, Pras M, Weiss DT, Solomon A. Micro-method to isolate and purify amyloid proteins for chemical characterization. Amyloid. 2001;8(1):22–9. doi: 10.3109/13506120108993811 11293822

[28] Wall JS, Paulus MJ, Gleason S, Gregor J, Solomon A, Kennel SJ. Micro-imaging of amyloid in mice. Methods Enzymol. 2006;412:161–82. doi: 10.1016/S0076-6879(06)12011-X 17046658 PMC1805492

[29] Stokes G. An improved Congo red method for amyloid. Med Lab Sci. 1976;33(1):79–80. 59298

[30] Pras M, Schubert M, Zucker-Franklin D, Rimon A, Franklin EC. The characterization of soluble amyloid prepared in water. J Clin Invest. 1968;47(4):924–33. doi: 10.1172/JCI105784 5641627 PMC297240

[31] Martin EB, Stuckey A, Powell D, Lands R, Whittle B, Wooliver C, et al. Clinical Confirmation of Pan-Amyloid Reactivity of Radioiodinated Peptide (124)I-p5+14 (AT-01) in Patients with Diverse Types of Systemic Amyloidosis Demonstrated by PET/CT Imaging. Pharmaceuticals (Basel). 2023;16(4). 10.3390/ph16040629 PMC10144944 37111386

[32] Gertz MA, Dispenzieri A. Systemic Amyloidosis Recognition, Prognosis, and Therapy: A Systematic Review. JAMA. 2020;324(1):79–89. doi: 10.1001/jama.2020.5493 32633805

[33] Frantellizzi V, Cosma L, Pani A, Pontico M, Conte M, De Angelis C, et al. Role of Nuclear Imaging in Cardiac Amyloidosis Management: Clinical Evidence and Review of Literature. Curr Med Imaging. 2020;16(8):957–66. doi: 10.2174/1573405615666191210103452 33081658

[34] Papantoniou V, Valsamaki P, Kastritis S, Tsiouris S, Delichas Z, Papantoniou Y, et al. Imaging of cardiac amyloidosis by (99m)Tc-PYP scintigraphy. Hell J Nucl Med. 2015;18 Suppl 1:42–50. 26665211

[35] Ehman EC, El-Sady MS, Kijewski MF, Khor YM, Jacob S, Ruberg FL, et al. Early Detection of Multiorgan Light-Chain Amyloidosis by Whole-Body (18)F-Florbetapir PET/CT. J Nucl Med. 2019;60(9):1234–9. doi: 10.2967/jnumed.118.221770 30954943 PMC6735282

[36] Hutt DF, Quigley AM, Page J, Hall ML, Burniston M, Gopaul D, et al. Utility and limitations of 3,3-diphosphono-1,2-propanodicarboxylic acid scintigraphy in systemic amyloidosis. Eur Heart J Cardiovasc Imaging. 2014;15(11):1289–98. doi: 10.1093/ehjci/jeu107 24939945

[37] Cleynhens J, Verbruggen A. Technetium-99m radiopharmaceuticals—Radiochemistry and radiolabeling. Reference Module in Biomedical Sciences. 2021.

