# Supplementary material for: Preclinical evaluation of Tc-99m p5+14 peptide for SPECT detection of cardiac amyloidosis
Source: PLoS One. 2024 Apr 5;19(4):e0301756. doi: 10.1371/journal.pone.0301756 (PMC10997057; doi:10.1371/journal.pone.0301756)
Supplement: S1 Fig — Slides were processed for autoradiography for 2 days. Binding of the radiolabeled peptide to amyloid was evidenced by the presence of black silver grains in the autoradiographs (ARG). The presence of higher density grains correlated with amyloid deposits seen as red fluorescence in the Congo red (CR)-stained consecutive tissue sections. (DOCX) [file pone.0301756.s001.docx]

**Supplementary Figures**

**Supplementary Figure 1**.

Formalin-fixed AL amyloid-laden kidney tissue sections were overlayed with ^99m^Tc p5+14 (~60 nCi, ~ 7 ng in 10 µL), incubated and washed as described in methods. Slides were processed for autoradiography for 2 days. Binding of the radiolabeled peptide to amyloid was evidenced by the presence of black silver grains in the autoradiographs (ARG). The presence of higher density grains correlated with amyloid deposits seen as red fluorescence in the Congo red (CR)-stained consecutive tissue sections.
